# A Study of the Soil–Wall–Indoor Air Thermal Environment in a Solar Greenhouse

**DOI:** 10.3390/s25134041

**Published:** 2025-06-28

**Authors:** Zhi Zhang, Yu Li, Liqiang Wang, Weiwei Cheng, Zhonghua Liu

**Affiliations:** 1 College of Agricultural Engineering, Shanxi Agricultural University, Jinzhong 030801, China; zhangzhi9170@163.com (Z.Z.); 13453047319@163.com (Y.L.); 15635994486@163.com (L.W.); 2 College of Urban and Rural Construction, Shanxi Agricultural University, Jinzhong 030801, China; lzh6175305@163.com

**Keywords:** sunken solar greenhouse, thermal environment (soil wall indoor air), coupling mechanism, LSTM model, marginal effect

## Abstract

Greenhouses offer optimal environments for crop cultivation during the winter months. The rationale for this study was identified as the synergistic exchange of air between the soil, the wall, and the indoor environment within the greenhouse (referring to the coupling law of the temperature fields of the three elements in space and time, including the direction of heat transfer and the consistency of the temperature zoning), thereby maintaining a more optimal temperature. However, there is a paucity of research on the impact of different spans on the thermal environment in solar greenhouses and even fewer studies on the synergistic law of changes in soil-wall indoor air in solar greenhouses with different spans. In this study, two solar greenhouses with different spans were analyzed through a combination of experiments as follows: K-means classification optimized using the grey wolf optimizer (GWO), computational fluid dynamics (CFD) simulations, and long short-term memory (LSTM) prediction models. The two solar greenhouses, designated as S1 and S2, had spans of 11 m and 10 m, respectively. The results are as follows: In two greenhouses when the span and temperature were the same, the indoor air temperature and soil temperature of the S1 greenhouse were lower than those of the S2 greenhouse; there was an isothermal layer in the north wall of greenhouses S1 and S2 (a stable area where the temperature change over time is less than 0.5 °C), the horizontal distance between the isothermal layer on the inside of the greenhouse wall and the inside of the wall was more than 400 mm, and that of the outside of the greenhouse wall was more than 200 mm; within the solar greenhouse, this study identified that heat was emitted from the inner surface of the wall (at 0 mm from the inner surface) toward the outer surface of the wall (at 0 mm from the outer surface), as well as at a horizontal distance of 200 mm from the inner surface of the wall. The temperature data from 0:00 to 8:00 at night were selected for the purpose of analyzing the temperature synergistic change in soil-wall indoor air in the S1 greenhouse. The temperature change can be classified into four categories according to K-means classification, which was optimized based on the grey wolf algorithm. The categories were as follows: high-temperature region, medium-high temperature region, medium-low temperature region, and low-temperature region. The low-temperature region spanned the range of X = (800, 3000) mm, and its height range was Y = (−150, 1200) mm. The CFD model and LSTM prediction model have been shown to be superior, and the findings of this study offer a theoretical basis for the optimization of thermal environment control in solar greenhouses.

## 1. Introduction

This study notes that the food crisis has affected the development of countries due to the changes in the world economic pattern, the Russian–Ukrainian conflict, and the turbulent situation in the world. As a typical energy-saving horticultural facility in China, solar greenhouses can grow vegetables and fruits and melons in winter, effectively alleviating the challenges of counter-seasonal vegetable production [1]. A greenhouse with a sunken earth wall is a kind of greenhouse with an original structural type in China whose outstanding advantages are low cost and good heat storage performance, which is very suitable for the actual situation of China’s rural areas with a wide geographic area and poor economic base [2]. Among them, the heat preservation effect of a sunken solar greenhouse is higher than that of an ordinary greenhouse [3].

The indoor temperature affects enzyme activity, which in turn affects the photosynthetic rates, growth, and the quality and morphology of crops [4], based on which a photosynthetic rate prediction model was developed to obtain the photosynthetic rate at different temperatures [5]. However, the traditional measurement methods were not only time-consuming and laborious but also it was also difficult to predict the temperature changes in the greenhouse in advance [6]. The group explored the indoor air temperature change mechanism through mathematical modelling [7], experimental studies, and CFD software 19.0 [8]. The study [9] showed that regardless of the weather conditions, the indoor air temperature changed more drastically along the vertical and horizontal directions, while the rate of change along the longitudinal direction of the greenhouse was more moderate. Based on this study, the group [10] further completed research on the marginal effect under solar greenhouse films and the results showed the following: during the wintering period, near the films there is a low-temperature region of approximately equal temperatures, and the south side of the lower corner of the maximum horizontal distance of 6130 mm, from the ground height of the minimum of 600 mm. The canopy temperature in the greenhouse is due to the crop growth, external light intensity, wall heat storage, covering materials, and other factors. The spatial distribution of the crop canopy temperature was non-linear and variable, which made it difficult to apply the mechanism model in long-term accurate prediction [11], and it was relatively difficult to simulate accurately using CFD technology [12]. Studies had been conducted to predict and study the air temperature in solar greenhouses using deep learning models [13], and most of the predictions were good. Arranging a large number of measurement points in the greenhouse and predicting and controlling the key measurement points in the greenhouse through deep learning models [14] have become hot topics for research on solar greenhouse regulation.

Soil temperature is an important environmental factor to ensure the growth of plant roots: a higher or lower soil temperature will affect the growth of plant roots, but less research is available on soil temperatures [15]. Concerning the distribution of soil temperature in the greenhouse [16], the relevant studies have shown that the south side of the indoor soil had a marginal effect and the marginal effect boundary point changed with time and seasonal variations [17]; in the overwintering period, the solar greenhouse soil thermal environment can be divided into a low-temperature zone, constant-temperature zone, and a high-temperature zone, with the low-temperature zone of the soil needing to be heated [10]; with an increase in soil depth, the rate of change in the soil temperature gradually approaches 0 [18]. The water content also affects the soil temperature [19], and low soil water content leads to higher soil temperatures; in particular, a shallow soil temperature was negatively correlated with the effect of the soil water content [20]. While continuous cropping in solar greenhouses demonstrated better soil properties than arable crops in the short term due to changes in irrigation and fertilization regimes, there was a potential risk of salinization and acidification [21]. Researchers have calculated the soil temperature and water content using partial differential equations [22]. The model accounted for radiative properties to calculate radiative fluxes, revealing a complex relationship between soil temperature changes and environmental variations both inside and outside the greenhouse, including the greenhouse envelope [23]. Additionally, it identified multiple heat-transfer modes at the soil boundary [24]. Building on this, researchers have used programming to develop a solar greenhouse thermal environment simulation based on VB and MATLAB 6.5, focusing on the stratified heat balance of greenhouse soil. Optimization studies have been conducted on solar greenhouse structures across different regions [25], aiding in the evaluation and selection of effective ground-temperature enhancement measures [26]. The prior research has primarily examined the relationship between soil temperature, depth, water content, and the southern margin area. However, the overall thermal environment involving indoor soil, wall, and night-time air temperatures remains unanalyzed.

The rear wall of a solar greenhouse consists of an insulation layer, a stabilization layer, and a heat storage layer [27]. Studies, both experimental [28,29,30,31] and via CFD simulations [32,33], have shown that the wall’s temperature varies horizontally and vertically [34]. Notably, when the wall exceeds 0.7 m in thickness, it consistently conducts heat outdoors at night. Following these studies, researchers have suggested alternative materials for heat storage, replacing the traditional earth wall, and have conducted corresponding experiments [35]. Recently, the construction of assembled solar greenhouses has increased, prompting research on heat storage in the rear walls of flexible designs [36]. Most studies have focused on the materials of the northern walls and temperature distribution. However, there is limited research on adjusting facilities to optimize heat absorption by the walls while ensuring crops receive maximum photosynthesis, thus maintaining the minimum temperature required for the greenhouse.

Numerous studies have explored temperature distribution within solar greenhouses. Previous research by our team has focused on indoor air temperature distribution and low-temperature areas under the greenhouse covering. However, these studies have typically examined isolated environmental factors or specific locations, lacking a comprehensive analysis of the thermal conditions affecting indoor air, soil, and walls in relation to plant growth. This study seeks to investigate the dynamics of the thermal environment (the soil-wall indoor air) in sunken solar greenhouses. Through a combined approach involving experimental investigations, numerical simulations, and LSTM prediction models, this research aims to establish a theoretical framework for enhancing the thermal environment regulation in solar greenhouses.

## 2. Experimental Methods and Materials

### 2.1. Experimental Materials

#### 2.1.1. Experimental Greenhouse

The greenhouse type was determined by employing the design parameters of a sunken solar greenhouse. The experimental greenhouses were located in the Taigu District of Jinzhong City, Shanxi Province, China, at coordinates 112.50° E longitude and 37.37° N latitude.

Greenhouse 1 had the following dimensions: a north–south span of 11 m and an east–west length of 76 m. The south side featured a trellis-covered arch, while the north wall tapered from a base width of 8 m to 2 m at the top over a sloping length of 4 m. The east and west walls mirrored the design of the north wall, with a width of 8 m at the base and 2 m at the top.

Greenhouse 2 had dimensions of 10 m in the north–south span and 76 m in the east–west length. Subsidence was not detected on the south side, which was built as a trellis over an arch. The north wall featured a base width of 8 m, tapering to 2 m at the top, with a sloping length of 3.8 m. The east and west walls mirrored the parameters of the north wall, with a base width of 8 m and a top width of 2 m.

Greenhouses S1 and S2, constructed with identical wall and shed membrane materials, varied only in span length (11 m for S1 and 10 m for S2). The structural design of both greenhouses was otherwise nearly identical, thereby maintaining a uniform set of experimental variables.

#### 2.1.2. Experimental Equipment

**Figure 1:**
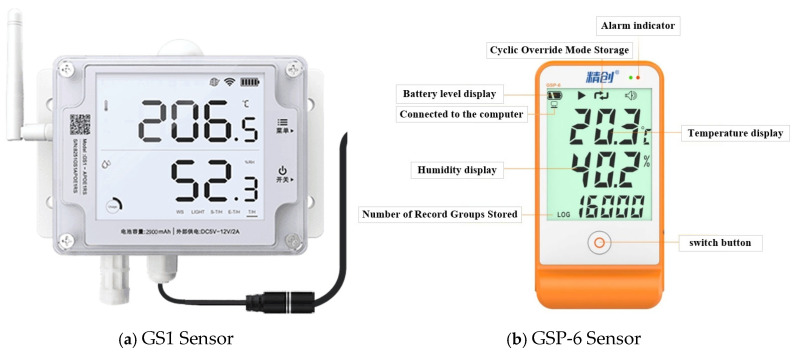
Experimental equipment.

In this study, the following instruments were used to assess air temperature, temperature, and light levels: an Easy Connect GS1 industrial-grade temperature and humidity recorder was employed to measure indoor light intensity and carbon dioxide concentration. The temperature recorder had a range of −20 to 60 °C with an accuracy of ±0.3 °C, while the humidity recorder had a range of 10–90% with an accuracy of ±3% RH. The light intensity recorder had a range of 0.01–157k lux with an accuracy of ±2%. The carbon dioxide concentration was measured in the range of −40,000 ppm, as depicted in Figure 1a.

Soil and wall temperature and humidity were assessed using the Jingchuang GSP-6 instrument, adhering to CE (EN12830), RoHS, and FDA (21CFR) guidelines. Data were collected every 10 min. The Jingchuang GSP-6 has a temperature range of −40 to 85 °C, with an accuracy of ±0.5 °C between −20 °C and +40 °C, and ±1 °C outside this range, as depicted in Figure 1b.

### 2.2. Methods of Analysis

#### 2.2.1. Experimental Methods

In their prior study [9], the researchers observed that the greenhouse’s thermal environment exhibited greater variability along both the vertical and horizontal axes. The central cross-section, positioned at the midpoint of the greenhouse in the east–west direction to exclude regions covered by cotton quilts, was selected. The experimental timeframe ranged from 24 December 2024 to 29 February 2025.

**Figure 2:**
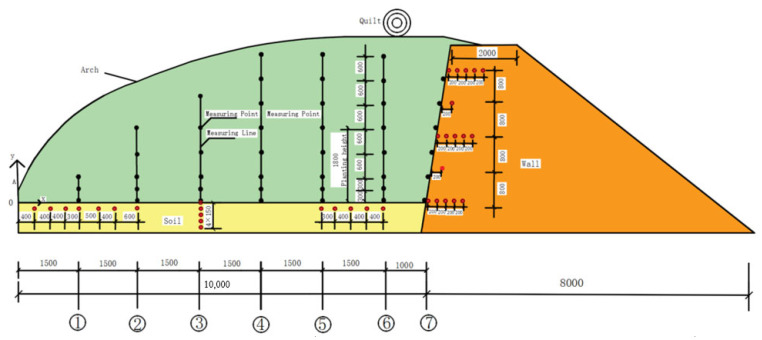
Layout of measurement points and lines.

The research team positioned air measurement points within the greenhouse as follows: Seven positions were selected along the central cross-section in the north–south direction. Pulleys were installed at these positions to suspend temperature sensors at varying heights. A total of 41 measurement lines were established, distributed across 7 lines. A coordinate system was defined with the southern base of the cross-section ridge as the origin, the y-axis pointing vertically upward, and the x-axis oriented northward. Line No. 1 was initiated at the southern edge of the melon planting area. Line No. 4 was positioned at the midpoint in the north–south direction, while Line No. 6 was placed at the northern end of the melon planting area. Line No. 7 was situated along the northern wall. The coordinates of the air measurement points are detailed in Figure 2 and Table 1.

In this investigation, soil measurement points were positioned as follows: At y = −150 mm, seven measurement points were distributed within the x range of (400, 3000), and five measurement points within the greenhouse x range of (7500, 9000). The spatial coordinates of the soil measurement points are illustrated in Figure 2 and detailed in Table 2. Within the S2 greenhouse, temperature measurement points were situated at x = 4500 mm, with corresponding y values of 0 mm, −150 mm, −300 mm, −450 mm, and −600 mm.

The wall measurement points were positioned at specific heights along the wall as follows: at y = 0 mm, 1800 mm, and 3600 mm, temperature and humidity measurements were taken at horizontal distances of 200 mm, 400 mm, 600 mm, and 800 mm from the inner surface of the north side of the wall. At y = 0 mm, 900 mm, 1800 mm, 2700 mm, and 3600 mm, temperature and humidity measurements were taken at a consistent horizontal distance of 200 mm from the inner surface of the wall. The exact coordinates of these measurement points can be found in Figure 2 and Table 3.

#### 2.2.2. Analysis of Variance (ANOVA)

Analysis of variance (ANOVA) is a statistical technique utilized to determine the statistical significance of mean differences among three or more independent sample groups. By partitioning the total data variability into between-group and within-group components, ANOVA computes the F-statistic to evaluate group differences. This approach relies on assumptions such as data normality, variance homogeneity, and observation independence. ANOVA serves as a reliable inferential method for comparing multiple groups and finds extensive application in disciplines like biostatistics, social sciences, and engineering.

To enhance data reliability, this study specifically analyzed data from measurement lines 2, 4, and 6, focusing on dynamic night-time temperature changes at three key time points: 22:00 on 28 January 2025 and 02:00 and 06:00 on 29 January 2025. A significance level of 0.05 was rigorously maintained for statistical inference. The findings of the analysis of variance (ANOVA) are detailed in the following table.

Table 4 displays *p*-values of 0.003, 0.020, and 0.041 at the three crucial night-time time points. These values fall below the predetermined significance threshold of 0.05, indicating statistical significance in temperature disparities among measurement lines. This study’s measurement techniques effectively differentiated temperature fluctuations across these lines, affirming the data collection precision and dataset reliability.

#### 2.2.3. K-Means Classification Based on the Grey Wolf Optimizer (GWO) Algorithm

This study utilizes the GWO-K-means algorithm, which combines the strengths of the grey wolf algorithm and the K-means algorithm. By leveraging the grey wolf algorithm to optimize the initial clustering center of the K-means algorithm, it mitigates the sensitivity of K-means to initial values and its tendency to converge to local optima. Theoretical analysis and empirical findings demonstrate the algorithm’s significant enhancements in clustering precision and robustness, offering a more efficient approach for clustering analysis.

The GWO-K-means algorithm integrates the grey wolf algorithm to identify optimal initial clustering centers for subsequent use in the K-means algorithm. The algorithm proceeds as follows: First, the grey wolf population is initialized, and N grey wolf individuals are randomly generated, each representing a set of initial clustering centers with dimensions k × d, where d denotes the data points’ dimensionality. The fitness value is calculated by employing each grey wolf individual as the initial clustering center for the K-means algorithm. Subsequently, the K-means algorithm is executed, with the objective function J serving as the fitness value for that individual. The grey wolf’s position is updated based on the grey wolf algorithm’s position update formula. Steps 2 and 3 are reiterated until the specified number of iterations is completed, and the individual with the optimal fitness value is chosen as the initial clustering center for the K-means algorithm. The K-means algorithm is then executed using the optimized initial clustering center to obtain the final clustering result.

##### K-Means Algorithm

The primary goal of the K-means algorithm is to minimize the sum of squared Euclidean distances between each data point and its respective cluster center. In other words, the algorithm aims to minimize the objective function.

$$\begin{array}{cc} J & =\sum_{i=1}^{k}\sum_{x_{j}\in C_{i}}∥x_{j}-\mu_{i}{∥}^{2}\\ \mu_{i} & =\frac{1}{n_{i}}\underset{x_{j}\in C_{i}}{\sum }x_{j} \end{array}$$

where k is the number of clusters; $C_{i}$ denotes the *i*th cluster; $x_{j}$ is the data points in the cluster; $\mu_{i}$ is the center of the i th cluster; and $n_{i}$ is the number of data points in cluster $C_{i}$.

##### The Grey Wolf Algorithm

In grey wolf packs, a strict social hierarchy exists, consisting of four main categories: the α-wolf, β-wolf, δ-wolf, and ω-wolf. The α-wolf typically represents the optimal decision-maker within the group, with the β-wolf and δ-wolf providing support in the decision-making process, while the ω-wolf follows the directives of higher-ranking wolves.

The position update formula in the grey wolf algorithm mimics the encircling behavior of grey wolves when hunting their prey:

$$\begin{array}{c} \vec{D}=\left|\vec{C}\cdot {\vec{X}}_{p}(t)-\vec{X}(t)\right|\\ \vec{X}(t+1)={\vec{X}}_{p}(t)-\vec{A}\cdot \vec{D} \end{array}$$

where t is the current iteration number; $\vec{X}$ is the current position of the grey wolf; $\vec{X_{p}}$ is the position of the prey; $\vec{A}$ and $\vec{C}$ is the coefficient vector, and the formula is as follows:

$$\vec{A}=2a\cdot {\vec{r}}_{1}-a,\;\vec{C}=2\cdot {\vec{r}}_{2}$$

where a decreases linearly from 2 to 0 and ${\vec{r}}_{1}$ and ${\vec{r}}_{2}$ are random vectors in the range [0,1].

$$\begin{array}{l} {\vec{D}}_{\alpha }=\left|{\vec{C}}_{1}\cdot {\vec{X}}_{\alpha }-\vec{X}\right|,{\vec{D}}_{\beta }=\left|{\vec{C}}_{2}\cdot {\vec{X}}_{\beta }-\vec{X}\right|,{\vec{D}}_{\delta }=\left|{\vec{C}}_{3}\cdot {\vec{X}}_{\delta }-\vec{X}\right|\\ {\vec{X}}_{1}={\vec{X}}_{\alpha }-{\vec{A}}_{1}\cdot {\vec{D}}_{\alpha },{\vec{X}}_{2}={\vec{X}}_{\beta }-{\vec{A}}_{2}\cdot {\vec{D}}_{\beta },{\vec{X}}_{3}={\vec{X}}_{\delta }-{\vec{A}}_{3}\cdot {\vec{D}}_{\delta }\\ \vec{X}(t+1)=\frac{{\vec{X}}_{1}+{\vec{X}}_{2}+{\vec{X}}_{3}}{3} \end{array}$$

where $X_{\alpha }$, $X_{\beta }$, and $X_{\delta }$ are the positions of the α wolf, β wolf, and δ wolf, respectively.

#### 2.2.4. CFD Numerical Simulation

To examine the impact of various spans on the synergistic variation in indoor soil–air–wall temperature in solar greenhouses during the extreme cold conditions in 2025, computational fluid dynamics (CFD) software version 19.0 was employed to simulate the night-time temperature distribution in S1 and S2 greenhouses. The simulation period spanned from 18:00 on 28 January 2025 to 8:00 on 29 January 2025, encompassing a lowest temperature of −11 °C. Subsequently, a meticulous partitioning of the simulation model was conducted, accurately assigning initial temperature values to different partitions based on both the simulation outcomes and measured data. This approach aimed to explore the impact of varying spans on the greenhouse temperature dynamics.

##### Greenhouse Model

**Figure 3:**
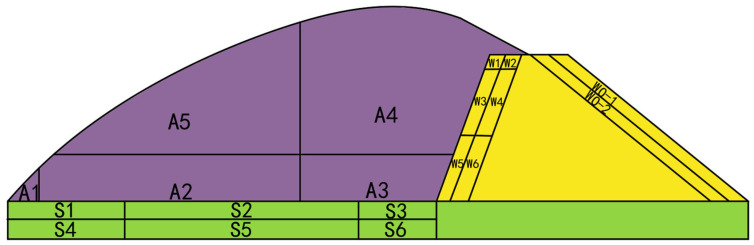
Greenhouse model.

The greenhouse model depicted in Figure 3 was discretized into 21 regions to enhance calculation precision. In this discretization, the air, soil, and wall regions were represented by the colors purple, green, and yellow, respectively. Within the greenhouse airspace, divisions were made at Y = 1200 mm and X = 800 mm, with 7500 mm serving as a boundary. This resulted in the subdivision of the airspace into five regions: A1, A2, A3, A4, and A5. Along the north wall of the greenhouse, divisions were established at X = 11,100 mm, 11,400 mm, 18,800 mm, 18,900 mm, Y = 1800 mm, and 3600 mm, creating nine regions: W1, W2, W3, W4, W5, W6, the north wall thermostat area, WO1, and WO2. The greenhouse soil was segmented into six regions, denoted as S1, S2, S3, S4, S5, and S6, with demarcations at 2000 mm and 8000 mm horizontally from the north wall of the greenhouse at Y = −150 mm and −600 mm.

##### Meshing

**Figure 4:**
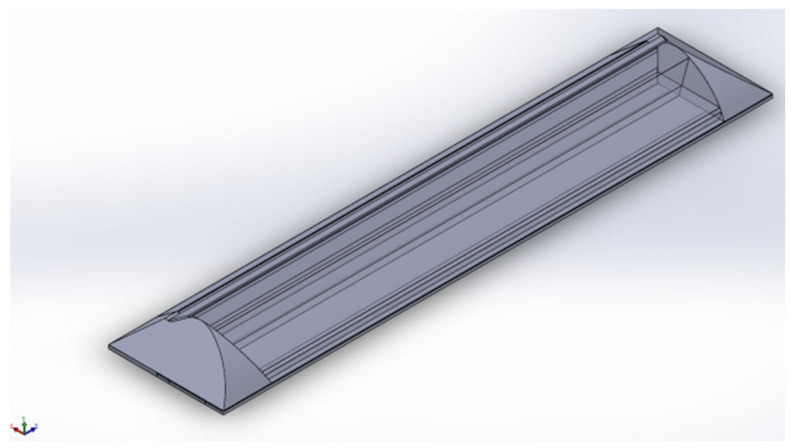
S1 and S2 greenhouse model.

**Figure 5:**
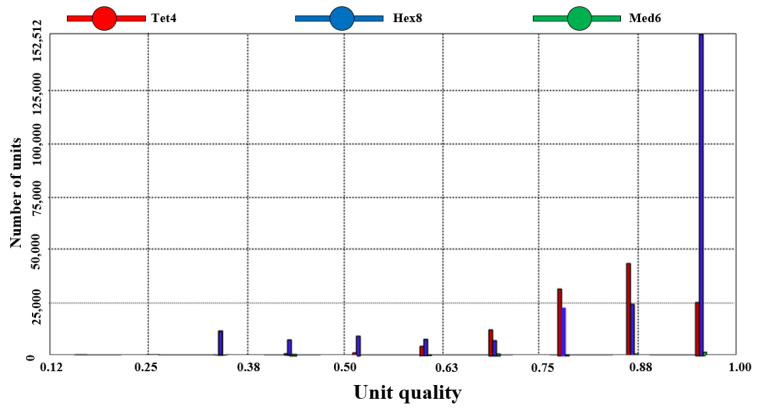
S1 greenhouse grid results and quality.

**Figure 6:**
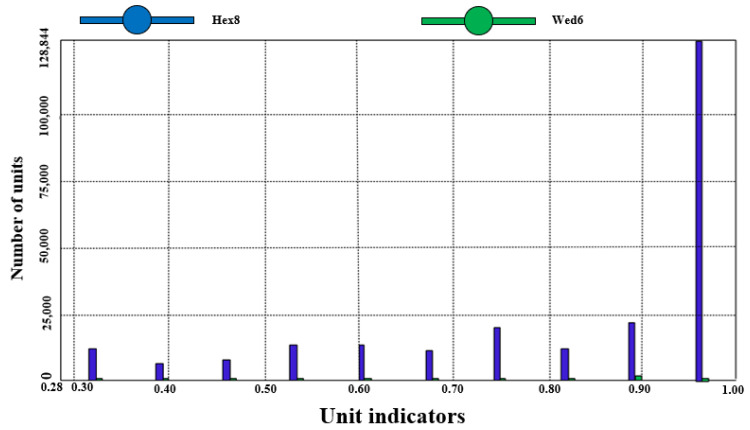
S2 greenhouse grid results and quality.

Figure 4 illustrates the creation of a 1:1 three-dimensional model of the solar greenhouse, subsequently imported into numerical simulation software. Interfaces were extracted using Boolean operations to address interfacial coupling. The model underwent meshing, with a grid cell size of 300 mm and a maximum internal size of 300 mm for both the S1 and S2 greenhouse 3D models. Figure 5 and Figure 6 depict the 3D model and its mesh quality post-meshing.

Gridding quality was assessed based on cell mass, revealing that over 70% of grids in both S1 and S2 greenhouses exhibited a cell mass of 0.88 or higher, and over 90% of grids were under 300 mm. The S1 model comprised 360,617 grid cells, while the S2 model comprised 242,736 grid cells, meeting the Fluent simulation test criteria by avoiding any “negative grid” errors.

To assess meshing rationality, a mesh sensitivity analysis was executed by conducting simulations with grid sizes of 300 mm and 200 mm. The findings indicated that refining the grid from 300 mm to 200 mm maintained the general temperature distribution trend, decreased temperature discrepancies in localized regions, and enhanced mesh quality. Specifically, with a 200 mm grid size, over 80% of grid cells exhibited a cell quality exceeding 0.88, and over 95% of cells were smaller than 200 mm. Nevertheless, to strike a balance between computational efficiency and precision, a 300 mm grid size was selected to ensure computational accuracy while managing computational expenses effectively.

##### Boundary Conditions and Initialization

To assess the impact of varying spans on the synergistic variation in indoor soil–air–wall temperatures, this study utilized data from the S1 greenhouse, characterized by a substantial north–south span, as the baseline dataset. Specifically, the temperature readings for each partition at 18:00 on January 28 were designated as the initial values. The average of these initial values for each partition is presented in Table 5. The initial temperature for the remaining boundary walls was standardized at 17 °C.

Suitable material parameters were chosen, and each thermophysical property of the materials was detailed in Table 6. Based on these properties, appropriate boundary conditions were established. The time frame from 18:00 on 28 January 2025 to 8:00 on 29 January 2025 was designated for the computational analysis across the entire domain to assess temperature variations in different regions over a 14-h period.

#### 2.2.5. LSTM Prediction Method

Long short-term memory (LSTM) is a sophisticated type of recurrent neural network architecture developed to mitigate the challenge of long-term dependencies faced by conventional RNNs in analyzing sequential data, particularly in scenarios involving extensive delays and prediction horizons. LSTM effectively enhances the model’s capability to process extended sequential information by incorporating three crucial gating mechanisms: an input gate, a forgetting gate, and an output gate, alongside a cell state. Within LSTM, the forgetting gate determines the information to be discarded from the cell state, the input gate identifies new information to be stored, and the output gate regulates how the cell state information influences the network’s hidden state.

Assuming the model input as (*x*1, *x*2, …, *xt*) and the hidden layer output as (*h*1, *h*2, …, *ht*), the LSTM receives inputs at time step *t* comprising the current network input *xt*, the previous hidden layer cell output *ht* − 1, and the previous cell state *ct* − 1. Through these inputs, the LSTM efficiently regulates the newly updated cell state. The input gate manages information input, the output gate controls information output, and the forgetting gate determines the retention of cell state history information.

$$ft=\sigma \left(Wf\cdot \left[ht-1,xt\right]+bf\right)$$

$$it=\sigma \left(Wi\cdot \left[ht-1,xt\right]+bi\right)$$

$$C∼t=\tanh\left(WC\cdot \left[ht-1,xt\right]+bC\right)$$

Ct=ft∗Ct−1+it∗C∼t

$$ot=\sigma \left(Wo\cdot \left[ht-1,xt\right]+bo\right)$$

$$ht=ot*\tanh\left(Ct\right)$$

In the equation provided, f signifies the activation state of the forgetting gate, i represents the activation state of the input gate, c indicates the cell state at the current time step, o denotes the activation state of the output gate, and ht signifies the output of the LSTM cell at time step *t*. Additionally, W represents the weight coefficient matrix, b signifies the bias term, t represents the time series, σ denotes the sigmoid activation function, and tanh denotes the hyperbolic tangent activation function.

Pre-processing plays a pivotal role in the analysis of experimental data by addressing outliers, missing values, and normalizing the data. This study employs an interpolation technique to replace outliers and missing values by averaging data from adjacent time points. Furthermore, to improve model accuracy and speed up convergence during training, data normalization is performed using the following formula:

$$X_{\mathrm{par}}=\frac{X-X_{\min}}{X_{\max}-X_{\min}}$$

where $X_{par}$ denotes the normalized data; X represents the original observations; $X_{\max}$ is the maximum value in the dataset; and $X_{\min}$ is the minimum value in the dataset.

In model evaluation, key indicators chosen to assess model performance include mean absolute error (*MAE*), mean square error (*MSE*), and coefficient of determination (R^2^). These metrics collectively gauge the accuracy and reliability of model predictions. The mean absolute error quantifies the average magnitude of the differences between predicted and actual values, while the mean square error represents the average of the squared differences. The coefficient of determination assesses the correlation between predicted and actual values. The formula for comparing predicted and actual values is expressed as follows:

$$\begin{array}{c} MAE=\frac{1}{n}\sum_{i=1}^{n}\left|\left(y_{i}-{\tilde{y}}_{i}\right)\right|\\ MSE=\frac{1}{n}\sum_{i=1}^{n}{\left(y_{i}-{\tilde{y}}_{i}\right)}^{2}\\ R^{2}=1-\frac{\sum_{i=1}^{n}{\left({\tilde{y}}_{i}-y_{i}\right)}^{2}}{\sum_{i=1}^{n}{\left({\bar{y}}_{i}-{\tilde{y}}_{i}\right)}^{2}} \end{array}$$

where $y_{i}$ denotes experimental data; $\tilde{y_{i}}$ denotes predicted values; and $\bar{y_{i}}$ is the mean value.

## 3. Results and Analysis

The solar greenhouse was specifically engineered to regulate indoor temperatures during night-time. To analyze its performance, data from the coldest day of the 2025 winter season, spanning from 17:00 on 28 January 2025 to 8:00 on 29 January 2025 was examined.

### 3.1. Analysis of the Indoor Air

**Figure 7:**
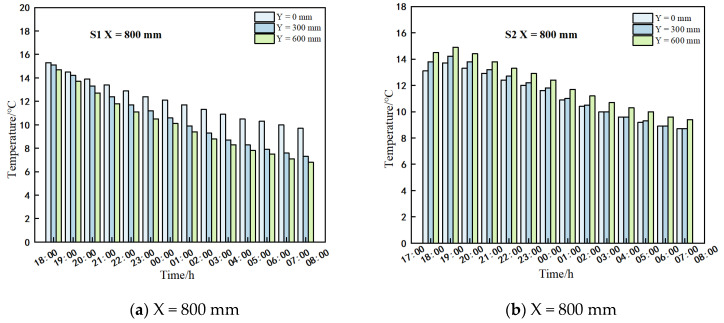

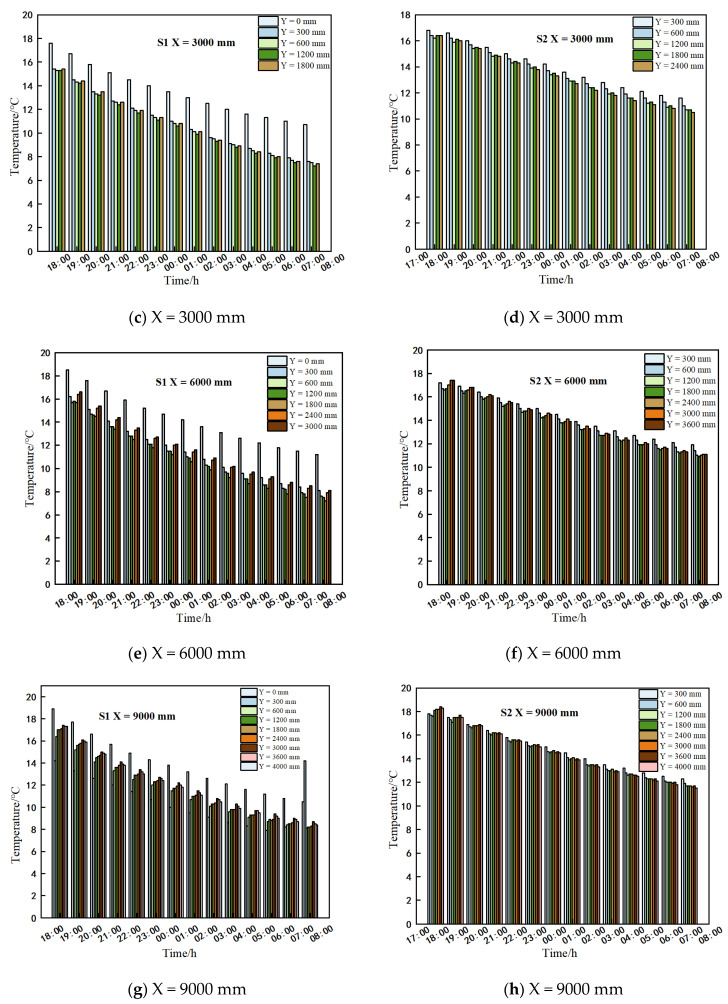
Temperature of each measurement line of S1 and S2.

Examination of Figure 7 reveals a consistent linear decline in air temperatures across all measurement lines within greenhouses S1 and S2 from 18:00 on 28 January 2025 to 8:00 on 29 January 2025. The temperature reduction range within the S1 greenhouse was recorded between 6.1 °C and 9.6 °C, whereas in the S2 greenhouse, it ranged from 3.1 °C to 7.3 °C. Notably, the temperature in the S2 greenhouse (spanning 10 m) exceeded that of the S1 greenhouse (spanning 11 m). In greenhouse S1, temperatures at the film and ground level of the same span were relatively higher during the day when the vents remained closed and at night after the cover film was sealed, while temperatures in the central region of the greenhouse were comparatively lower. A distinct observation was the more rapid temperature fluctuation at the top of the greenhouse during nocturnal temperature variations, with the upper temperature consistently remaining higher than that in the central area. Consequently, it is deduced that to elevate the night-time temperatures in the crop cultivation zone during winter nights, heating interventions should be targeted towards the lower section of the greenhouse.

Our research [9,10] has revealed a marginal cooling effect near the greenhouse canopy edge, creating a low-temperature zone of similar width within the greenhouse. To explore these marginal effects across varying widths within the greenhouse during night-time, we excluded data from soil surface measurement points to focus on cooler night-time air temperatures. Specifically, air temperature data from 0:00 to 8:00 were analyzed. Using the k-means classification method optimized with the grey wolf algorithm (GWO), we categorized temperature data from different heights within the same span and from the same height across different spans in greenhouses S1 and S2. The resulting low-temperature boundary points for corresponding measurement lines and heights are detailed in Table 7 and Table 8.

Table 7 displays varying low-temperature boundary heights for measurement lines in greenhouses S1 and S2 during the 0:00–8:00 period, depending on span heights. Despite this variability, the majority of low-temperature boundary points in both greenhouses clustered around 120 mm. Notably, the average temperature of these boundary points in greenhouse S2 exceeded that of S1, with temperature differentials ranging from 1.9 to 3.6 °C.

Table 8 displays distinct low-temperature boundary points for spans of varying heights in S1 and S2 greenhouses. Notably, the low-temperature boundary points differed between the two greenhouse types, predominantly clustering around the 7500 mm span. Within this span, the average temperature of the low-temperature boundary point in S2 greenhouses significantly exceeded that of S1 greenhouses by 1.0 to 3.3 °C.

In essence, the S1 and S2 greenhouses exhibited low-temperature zones ranging from 0 to 7500 mm in length and 300 to 1200 mm in height. It is imperative to monitor the air temperature in these zones during winter nights.

### 3.2. Changes in Soil Temperature

The findings from our 2024 study [9,10] revealed consistent soil temperature variations at specific locations (x = 1400 mm, 4600 mm, 8400 mm, and x = −150 mm vs. −350 mm) across the horizontal expanse of the greenhouse. Building upon these observations, the current investigation selected x = −150 mm as the reference depth for comparative analyses among greenhouses of varying widths. To establish a comprehensive vertical temperature profile, five temperature measurement points were strategically positioned at y = 5000 mm, x = 0 mm, −150 mm, −300 mm, −450 mm, and −600 mm within greenhouse 2. This approach aimed to systematically explore the soil temperature distribution patterns at standard depths in greenhouses of different widths.

#### 3.2.1. Soil Temperature at Different Depths

**Figure 8:**
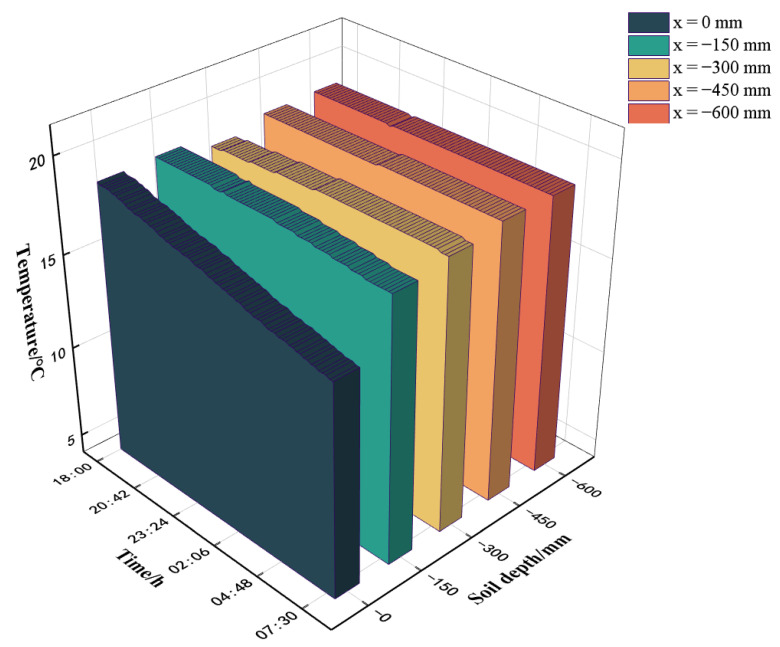
Soil temperature at different depths at y = 5000 mm in greenhouse 1.

Figure 8 illustrates a notable constraint on the greenhouse soil temperature at y = −150 mm within greenhouse 2, exhibiting substantial variability from y = 0 to −150 mm, while maintaining stability at y = −300 to −600 mm, consistent with the prior research by our team. In greenhouse 1, at x = 5000 mm, significant disparities in soil temperature were observed among different depths, particularly between y = 0 mm and y = −150 mm, with a maximum temperature difference of 2.7 °C. From 18:00 to 8:00 the following day, temperature reductions at the same location were 3.3 °C and 0.7 °C, respectively. Consistency in soil temperature patterns was observed at y = −300 mm, −450 mm, and −600 mm, with maximum temperature deviations from y = −150 mm of 0.6 °C, 0.8 °C, and 0.8 °C, respectively. Over the overnight period, temperature increases at these locations were 0.3 °C, 0.1 °C, and 0 °C, respectively. Thus, investigating the distribution of soil temperature specifically at y = −150 mm in the greenhouse is of paramount importance.

#### 3.2.2. Soil Temperatures at Different Spans

**Figure 9:**
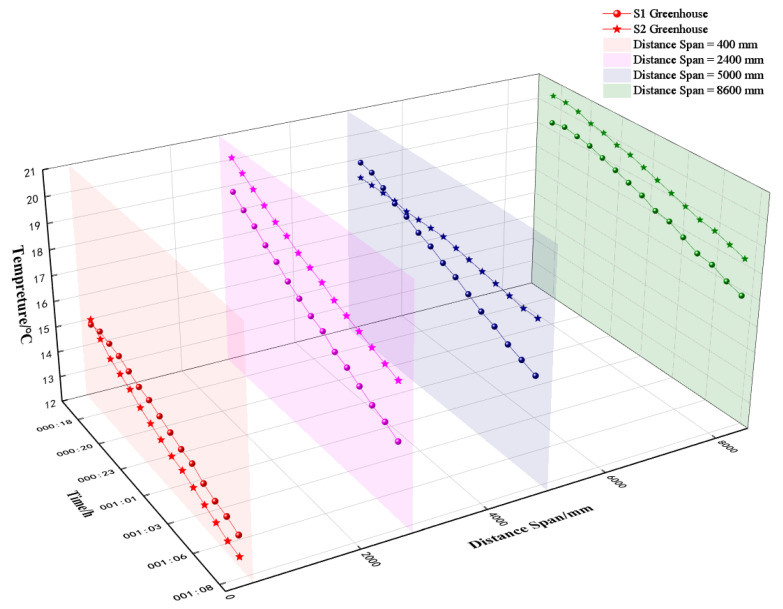
Soil temperature at different spans at x = −150 mm in greenhouses 1 and 2.

Figure 9 (different shapes and colors represent the distance span for greenhouse S1 and greenhouse S2, respectively) illustrates the variation in soil temperatures between greenhouse S1 and greenhouse S2. Both greenhouses exhibited a gradual night-time temperature decrease with increasing distance from the north wall. Notably, S1 consistently showed lower soil temperatures compared to S2 at equivalent spans and depths. This disparity can be attributed to the larger span of greenhouse S1, which resulted in a 1000 mm greater horizontal distance from the north wall compared to greenhouse S2. Consequently, greenhouse S1 experienced lower near-surface air temperatures at night, leading to correspondingly lower night-time soil temperatures within the crop-growing area. The presence of a 500 mm deep brick wall structure at the front end of greenhouse S1 reduced heat exchange between the front-side soil and the outdoor environment, resulting in higher soil temperatures at the front compared to greenhouse S2. Temperature measurements taken at specific points within the greenhouses revealed maximum temperatures in greenhouse S1 at x = −150 mm, y = 400 mm, 2800 mm, 5000 mm, and 8600 mm to be 15.4 °C, 19.2 °C, 19.3 °C, and 19.4 °C, respectively, from 18:00 to 8:00 the following day. These values corresponded to temperature decreases of 2.1 °C, 4.3 °C, 3.4 °C, and 2.5 °C, respectively. In comparison, greenhouse S2 recorded maximum temperatures of 15.5 °C, 20.5 °C, 18.7 °C, and 20.4 °C, with temperature decreases of 3.1 °C, 3.4 °C, 0.7 °C, and 2.2 °C at the same measurement points during the same time period. The maximum temperature differences between the two greenhouses at corresponding measurement points ranged from 1.8 °C to 2.3 °C.

A negative correlation was found between greenhouse soil temperature and its north-south span. Additionally, a significant disparity in soil temperature was observed between the front and middle sections of the greenhouse. Therefore, the implementation of a precise zoning strategy for greenhouse soil is crucial to optimize the utilization of soil heat resources in solar greenhouses.

#### 3.2.3. Cubic Function Fitting

This study analyzed greenhouse soil temperature data collected at a depth of y = −150 mm between 00:00 and 08:00. A cubic function curve was fitted to the average soil temperature data at each measurement point. Critical temperature values were identified at points where the slope of the cubic function was zero. The difference between the maximum and minimum critical temperature values was within 0.5 °C. The minimum critical temperature value was used to establish the boundary between the low-temperature zone and the constant-temperature zone in the first rising segment of the cubic function. Similarly, the maximum critical temperature value determined the boundary between the high-temperature zone and the constant-temperature zone in the second rising segment of the cubic function.

**Figure 10:**
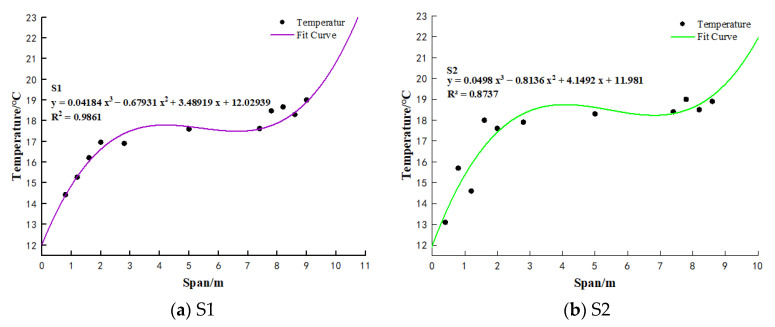
Soil temperature fitting curve of S1 and S2.

Figure 10 illustrates the temperatures recorded in greenhouse 1 and greenhouse 2. At a distance of 2 m from the north wall, greenhouse 1 and greenhouse 2 registered temperatures of 18.9 °C and 18.6 °C, respectively, while at 8 m from the north wall, the temperatures were 17.5 °C and 17.4 °C, respectively. In greenhouse 1, the temperature demarcation points for the soil’s low-temperature and constant-temperature zones were at y = 2970 mm and y = 7853 mm, corresponding to average soil temperatures of 17.5 °C and 17.8 °C, respectively. In greenhouse 2, these demarcation points were at y = 2660 mm and y = 8130 mm, corresponding to average soil temperatures of 18.2 °C and 18.7 °C, respectively. The north–south spans of greenhouse 1 and greenhouse 2 were 11 m and 10 m, respectively. Comparatively, greenhouse 1 had a smaller soil thermostatic zone than greenhouse 2, with lower temperatures. Notably, both the extent and temperature of the greenhouse soil’s constant-temperature zone decreased as the north–south span of the greenhouse increased.

In essence, the soil within the greenhouse can be categorized into two vertical zones: 0 mm < y < −150 mm and −150 mm < y < −600 mm. It can be classified into three horizontal regions: low-temperature, constant-temperature, and high-temperature zones. Increasing the north-south span of the greenhouse leads to a reduction in the extent of the constant-temperature zone within the soil. Therefore, implementing partition control of distinct soil regions within the greenhouse is crucial for optimizing energy utilization efficiency.

### 3.3. Changes in Wall Temperature

The temperature distribution of a solar greenhouse wall significantly influences the microclimate within the facility. This study focused on analyzing temperature data collected between 18:00 on 28 January 2025 and 8:00 on 29 January 2025. The investigation compared temperatures at varying heights (0, 3600 mm) along the walls of two types of solar greenhouses: a sunken solar greenhouse (S1) and an ordinary solar greenhouse (S2). These greenhouses differed in their horizontal depths (0, 800 mm) from the wall surface. The sunken solar greenhouse (S1) had a slope height of 4000 mm, while the ordinary solar greenhouse had a slope height of 3800 mm. The W1 section was located 400 mm and 200 mm away from the slope heights of the S1 and S2 greenhouses, respectively. The W5 section, situated at the lowest point of the greenhouse, was in direct contact with the soil surface. This study aimed to explore the distribution characteristics of wall temperatures in different types of solar greenhouses and identify the factors influencing these temperature patterns.

#### 3.3.1. Temperature Changes in Indoor and Outdoor Walls of Solar Greenhouses

**Figure 11:**
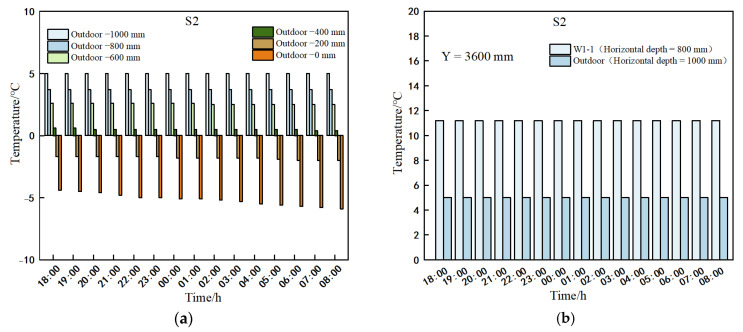
Comparison of horizontal depths of daylight greenhouse walls indoors and outdoors from the wall surface. (**a**) Temperature variation with time at different horizontal distances from the outdoor wall surface; (**b**) temperature at a horizontal distance of 1000 mm from the surface of the outdoor wall and temperature at a horizontal distance of 800 mm from the surface of the indoor wall as a function of time.

Figure 11 illustrates a gradual increase in the outdoor wall temperature with the increasing horizontal distance from the wall surface. Between 18:00 and 8:00 the following day, when the horizontal distance exceeded 200 mm, the average temperature difference across various wall depths was less than 0.3 °C, falling within the equipment’s measurement error range. Consequently, this depth can be considered the steady-state boundary of the wall temperature distribution. Model simulations indicate a decreasing trend in the outdoor surface temperature of the wall (at 0 mm) over time, with a decline of 1.5 °C. Meanwhile, temperatures remained constant at 800 mm from the indoor wall surface and 1000 mm from the outdoor wall surface, with a consistent temperature difference of 6.2 °C between them.

#### 3.3.2. The Air Temperature on the Interior Wall Surface and the Soil Temperature on the Inner Surface of the Wall

**Figure 12:**
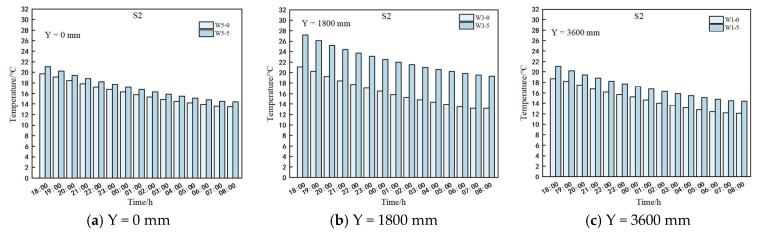
Comparison of temperatures at the inner surface of the wall at different heights and at 0 mm from the outer surface.

Figure 12 depicts the temperatures at various heights denoted as W5-0, W3-0, and W1-0 positioned horizontally at 0 mm from the outer surface of the inner wall of the solar greenhouse. Similarly, W5-5, W3-5, and W1-5 represent temperatures at different heights, located 0 mm horizontally from the inner surface of the inner wall. As depicted in Figure 1, temperatures at the inner surface of the wall at 0 mm consistently exceeded those at the outer surface between 18:00 and 8:00 the following day. Both inner and outer surface temperatures exhibited a declining trend over time, indicating heat transfer from the inner surface to the outer surface of the wall within the greenhouse.

#### 3.3.3. Indoor Wall Temperature Along the Transverse Law of Change

**Figure 13:**
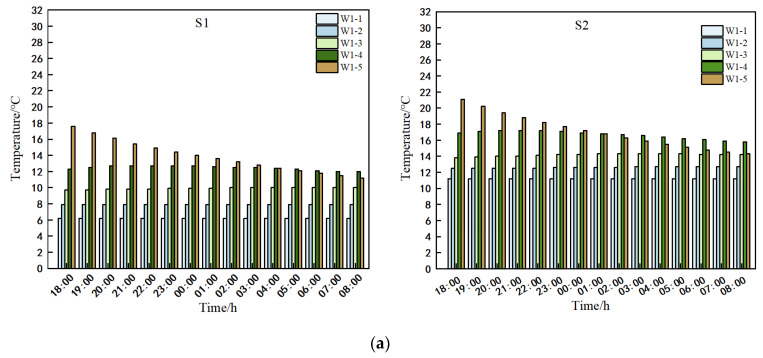

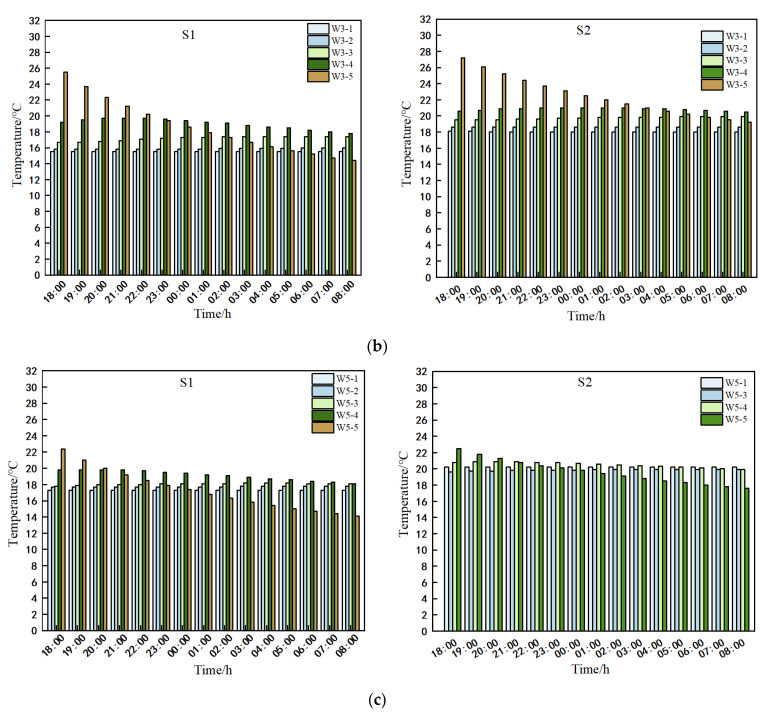
Transverse temperature variation in measured lines at different heights of the wall. (**a**) Plot of temperature versus time for different horizontal distances from the wall surface at Y = 3600 mm. (**b**) Plot of temperature versus time for different horizontal distances from the wall surface at Y = 1800 mm. (**c**) Plot of temperature versus time for different horizontal distances from the wall surface at Y = 0 mm.

Figure 13 illustrates that the S2 greenhouse consistently maintained a higher temperature compared to the S1 greenhouse at the W1 and W3 interfaces. This temperature difference can be attributed to the S1 greenhouse’s lack of open air vents and the obstruction of solar radiation by quilts on the wall. In contrast, the S2 greenhouse had open air vents and unobstructed solar radiation on the wall. Therefore, adjusting the position of the quilts during the daytime is recommended to optimize heat absorption by the wall. In the W5 interface, ventilation had minimal impact on greenhouse temperatures. Initially, the wall surface temperature represented the maximum temperature for both greenhouses. Technical inaccuracies suggest that temperature fluctuations below 0.5 °C should be considered within a temperature steady state.

Between 18:00 and 8:00 the following day, temperature measurements at points W1-5, W3-5, and W5-5 in sections W1, W3, and W5 of greenhouses S1 and S2 exhibited a consistent downward trend, showing the most significant temperature decrease. In the S1 greenhouse, temperatures decreased by 6.4 °C, 11.1 °C, and 8.3 °C, while in the S2 greenhouse, temperatures decreased by 6.9 °C, 8.1 °C, and 4.9 °C. Measurements at points W1-4 and W3-4 in sections W1 and W3 of both S1 and S2 greenhouses displayed a pattern of initial increase followed by decrease, reaching a point of temperature equilibrium with W1-5 and W3-5 before declining. During this period, the S1 greenhouse experienced temperature changes of 0.4 °C and 1.4 °C, while the S2 greenhouse saw changes of 1.4 °C and 0.5 °C. The temperature at W5-4 in the W5 section of the S1 greenhouse initially stabilized and equilibrated with W5-5 before decreasing by 1.7 °C. Conversely, the temperature at W5-4 in the W5 section of the S2 greenhouse initially rose to match W5-5 before decreasing by 1.0 °C over the same timeframe.

The wall surface temperatures of sections W1, W3, and W5 in greenhouse S1 matched the temperature at a depth of 200 mm horizontally at 4:00, 22:00–23:00, and 20:00–21:00, respectively. Between 18:00 and the flat time period, W1-5, W3-5, and W5-5 decreased by 5.2 °C, 5.3 °C, and 2.4 °C, while W1-4, W3-4, and W5-4 increased by 0.4 °C, 0.5 °C, and 0 °C. Similarly, in greenhouse S2, the wall surface temperatures of sections W1, W3, and W5 aligned with the temperature at a 200 mm depth at 1:00, 3:00–4:00, and 20:00–21:00. During the same 18:00 to flat time period, W1-5, W3-5, and W5-5 decreased by 4.4 °C, 6.6 °C, and 1.2 °C, respectively, while W1-4, W3-4, and W5-4 increased by 0.3 °C, 0.4 °C, and 0.1 °C. These observations indicate the heat transfer from the inner surface of the wall at 0 mm to a horizontal depth of 200 mm in the solar greenhouse.

Between 18:00 and 6:00 the following day in the S1 greenhouse, temperatures remained below 0.4 °C when the horizontal depth of the W1 section from the wall surface exceeded 200 mm, establishing a steady-state threshold for the W1 section. At a horizontal depth of 400 mm from the wall surface, the W3 and W5 sections exhibited a slight temperature increase of less than 0.5 °C. Consequently, the steady-state limit for wall temperature was set at a depth exceeding 400 mm for W3 and W5 sections, defining the boundary point for the steady-state wall temperature. In the S2 greenhouse, temperatures showed a minor increase of less than 0.5 °C when the distance of the W1 and W5 sections from the wall surface exceeded 400 mm, designating this distance as the steady-state boundary point for wall temperature. Similarly, in the W3 section, temperatures increased slightly when the horizontal depth from the wall surface surpassed 200 mm, with temperature changes below 0.5 °C. Therefore, the W3 section was identified as the steady-state boundary point for wall temperature at a horizontal depth exceeding 200 mm. Detailed findings are presented in Table 9.

#### 3.3.4. Vertical Temperature Changes in Indoor Walls in Different Types of Solar Greenhouses

**Figure 14:**
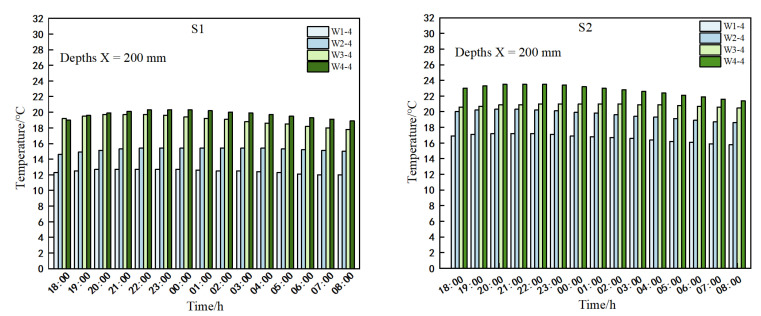
Temperature variation with time at different heights at a horizontal distance of 200 mm from the interior wall.

As depicted in Figure 14, the highest temperature was recorded at W4-4 due to direct sunlight exposure. Concurrently, temperatures at measurement points within greenhouses S1 and S2 exhibited a gradual decline with increasing height. Between 18:00 and 8:00 the following day, vertical temperature profiles in both greenhouses displayed an ascending–descending trend, peaking at W4-4 (notably, the temperature difference between W3-4 and W4-4 at 18:00 in greenhouse S1 was 0.2 °C). In greenhouse S1, the average temperatures at W4-4, W3-4, W2-4, and W1-4 were 19.8 °C, 19.1 °C, 15.2 °C, and 12.5 °C, respectively. Similarly, in greenhouse S2, the corresponding average temperatures were 22.7 °C, 20.9 °C, 19.6 °C, and 16.7 °C. Notably, at a horizontal distance of 200 mm from the internal wall surface at the same height, the temperature in S2 exceeded that in S1. This disparity can be attributed to the upwind vents being open in greenhouse S2, causing a backward shift in the quilt position and enabling the wall with a greater vertical distance from the ground in S2 to receive direct solar radiation.

In essence, the heat transfer within the solar greenhouse occurred from the inner surface of the outer wall to the outer surface of the inner wall, spanning a horizontal depth of 200 mm. A stable temperature layer was observed at distances exceeding 400 mm above the inner wall surface in both greenhouse S1 and S2. The steady-state boundary for the outer wall extended beyond 200 mm from its surface. Notably, the placement of the insulation significantly influenced the vertical temperature distribution within the greenhouse.

### 3.4. K-Means-Based Synergistic Change Analysis

The S1 greenhouse experienced low temperatures prompting an investigation into the combined temperature variations of soil, walls, and indoor air. To analyze this, the coldest days during January–March 2025, namely 29 January, 8 February, 24 February, and 5 March, were selected. Temperature data from 0:00 to 8:00 were specifically chosen for analysis. The K-means classification method, enhanced by the grey wolf algorithm, was employed for categorization. An elbow diagram indicated a distinct inflection point for a four-category division, signifying its optimal suitability. Subsequently, synergistic zoning and contour maps for these four days were generated, depicted in the accompanying figure.

**Figure 15:**
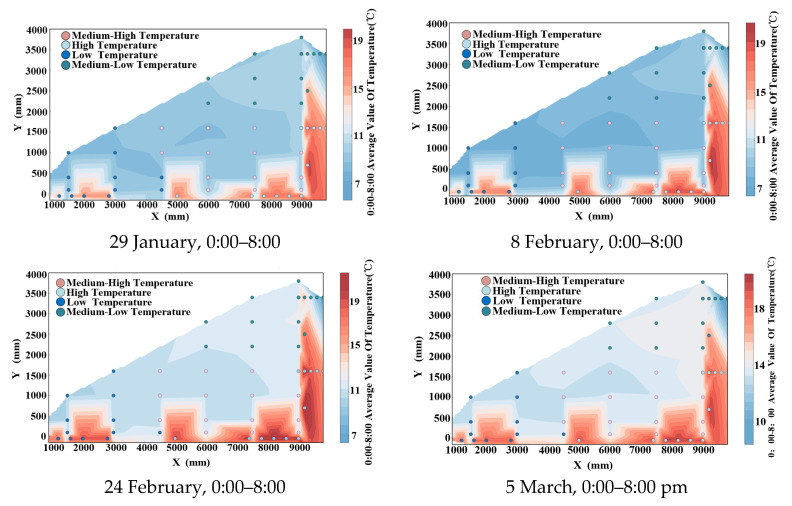
January-March 2025 synergy zone map and contour map.

Figure 15 illustrates the partitioning of soil–wall–indoor air temperature in the greenhouse between 0:00 and 8:00 into distinct zones of varying temperatures: high, medium-high, medium-low, and low. Despite lower temperatures observed near the trellis, the overall average temperature of the soil and wall was elevated, placing them in the high-temperature zone. Specifically, the low-temperature zone spanned X = 800 mm–3000 mm and Y = −150 mm–1200 mm, the medium-high temperature zone spanned X = 3000 mm–9000 mm and Y = 0 mm–1200 mm, and the medium-low temperature zone spanned X = 6000 mm–9000 mm and Y = 1200 mm–4000 mm.

During the 0:00–8:00 time period, the soil and walls exhibited high temperatures based on the mean temperature of the contour lines. Proximity to the soil and walls corresponded to the medium-high temperature zone, while areas closer to the walls but farther from the soil were categorized as medium-low temperature zones. Conversely, locations farther from the walls but nearer to the soil were classified as low-temperature zones.

Given that the crop layer is less than 2 m in height, our analysis indicates that heating should primarily occur during night-time in regions with low to medium temperatures, with particular attention to elevating temperatures in areas with lower temperatures.

### 3.5. Co-Analysis Based on CFD

To examine the impact of varying spans on soil temperature, wall temperature, and indoor air temperature under uniform working conditions, computational fluid dynamics software version 19.0 was utilized to model the S1 and S2 greenhouses. Specifically, temperature data from different zones within the S1 greenhouse at 18:00 on 28 January 2025 were chosen for simulation, with initial values detailed in Table 4.

#### 3.5.1. Model Correctness Analysis

**Figure 16:**
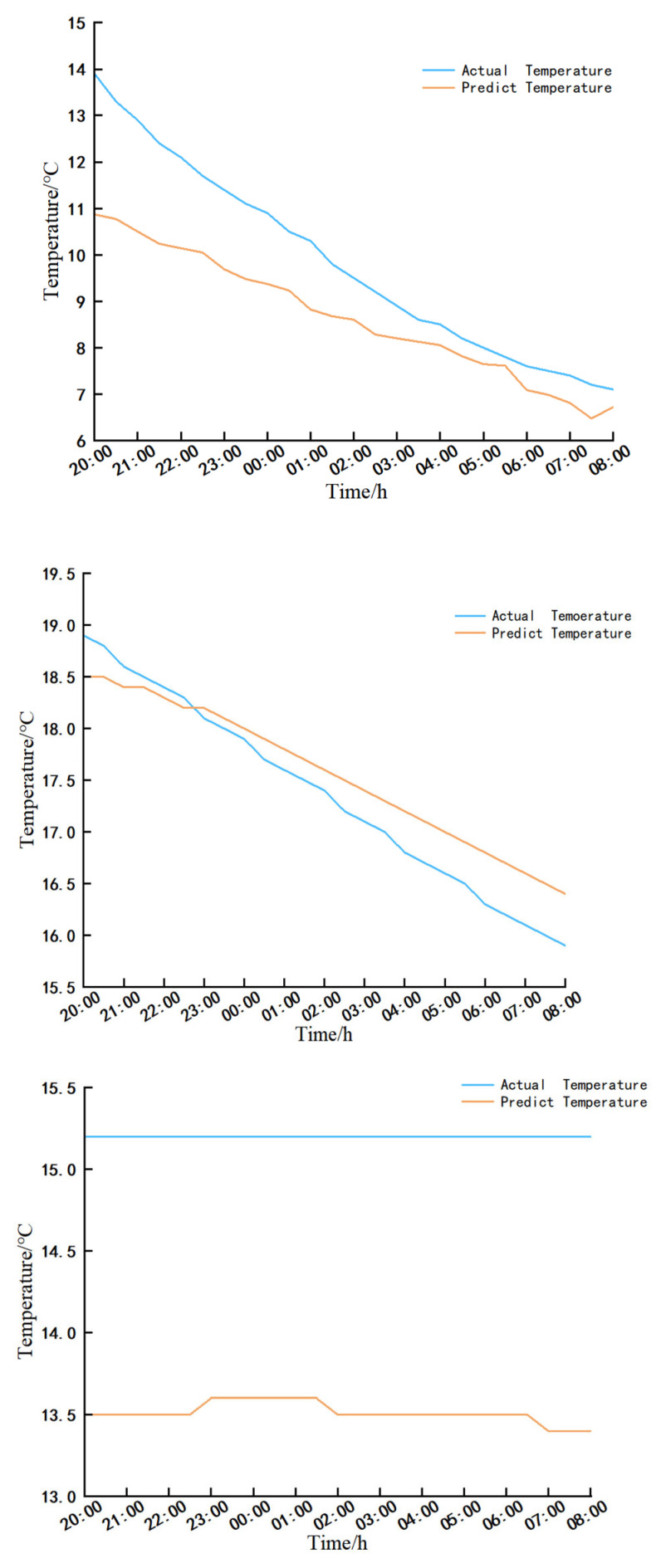
Comparison of predicted and actual data at measurement points (6000, 1800) (5000, −150), (11,500, 1800).

To validate the model’s accuracy, the S1 greenhouse was chosen. Predicted data from the CFD 19.0-post software for three measurement points (6000, 1800), (5000, −150), and (11,500, 1800) within the greenhouse were compared with actual measurements for the time span from 20:00 on 28 January 2025 to 8:00 on 29 January 2025. The comparison is illustrated in Figure 16, revealing maximum temperature deviations of 1.9 °C, 0.5 °C, and 1.8 °C, and minimum discrepancies of 0 °C, 0.1 °C, and 1.6 °C at the respective points. The absolute errors do not exceed 15%, indicating that the model’s errors are manageable. Thus, the numerical simulation results offer valuable insights.

#### 3.5.2. Influence of Different Spans on the Thermal Environment of the Greenhouse

To comprehensively analyze the temperature distribution patterns of solar greenhouses, cloud diagrams of S1 and S2 greenhouses were selected for analysis at 18:00 on 28 January 2025 and at 0:00, 4:00 and 8:00 on 29 January 2025.

##### Analysis of Air Temperature Simulation Results

**Figure 17:**
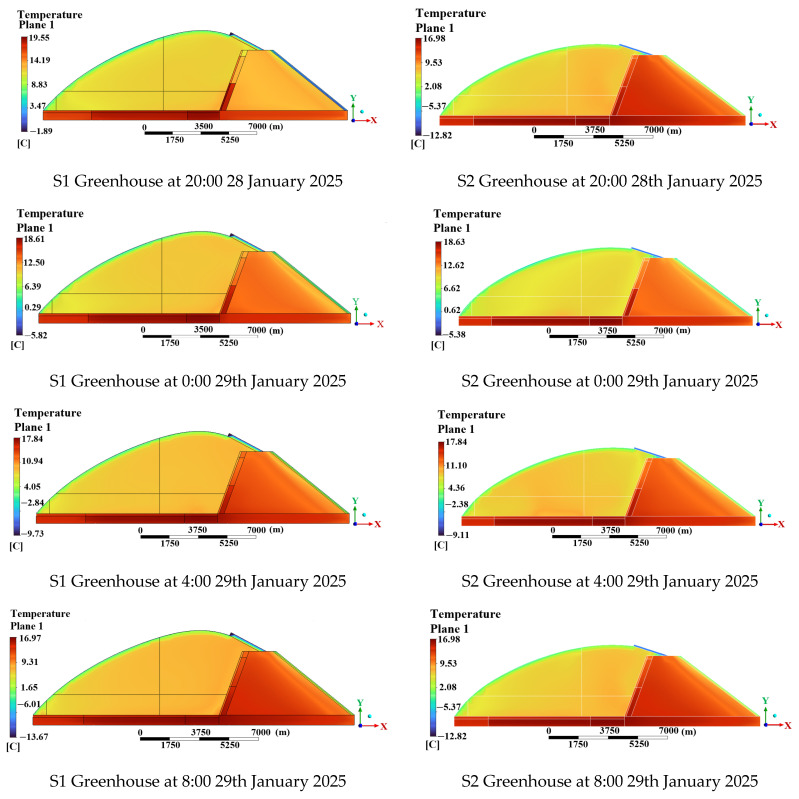
Temperature clouds at different moments in S1, S2 greenhouses.

Figure 17 illustrates a consistent linear decrease in temperature at the measuring points AF1 (7500, 1200) mm and AF2 (7500, 2400) mm within greenhouses S1 and S2 from 18:00 to 8:00 the following day. The temperature differentials between S1 and S2 for AF1 and AF2 ranged from a maximum of 0.57 °C to a minimum of 0.01 °C for AF1, and from 0.45 °C to 0.02 °C for AF2, respectively. These findings indicate that the differing widths of the greenhouses impact the temperatures at both AF1 and AF2.

##### Analysis of Soil Temperature Simulation Results

The figure above illustrates the following findings: between 18:00 and 8:00 the next day, a linear decrease in measurements was observed at measuring point SF1 (5000, −100) mm in greenhouse S1 and measuring point SF2 (5000, −500) mm in greenhouse S2. Concurrently, the temperature in greenhouse S2 exceeded that in S1, with temperature differentials of 0.1 °C and 0.09 °C, and minimal variances of 0 °C and 0.01 °C, respectively. These results suggest that the large-span greenhouse had a more pronounced impact on the SF2 measurement points, particularly on SF1 measurements.

##### Analysis of Wall Temperature Simulation Results

The figure above illustrates that between 18:00 and 8:00 the following day, there was a linear decrease in temperature at measuring points WF1 (400, 1000) mm and WF2 (6700, 1000) mm in greenhouses S1 and S2. Additionally, the temperature at WF1 was higher in greenhouse S2, while the temperature at WF2 was higher in greenhouse S1. The maximum temperature differences were 0.26 °C and 0.2 °C, respectively, while the minimum temperature differences were 0.04 °C and 0 °C, respectively. Hence, the greenhouses with varying span widths impact both WF1 and WF2 temperatures.

##### Analysis of Synergistic Wall–Soil–Indoor Air Changes in Greenhouses of Different Spans

To enhance the intuitive analysis of synergistic changes in wall–soil–indoor air interactions in greenhouses of varying spans, representative measurement points were designated as WF1 (400, 1000) mm on the wall, SF1 (5000, −100) mm on the soil, and AF1 (7500, 1200) mm on the indoor air. These points were positioned at the midpoint of greenhouse spans S1 and S2. Temperature change curves were then generated to depict temporal variations.

**Figure 18:**
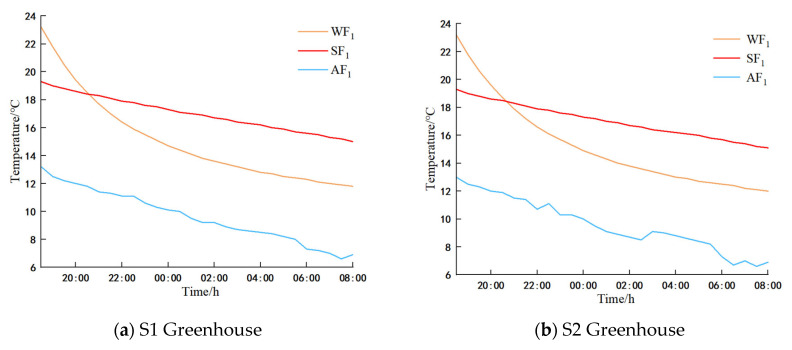
Wall–soil–indoor air temperature plots over time.

Figure 18 illustrates a notable simultaneous variation in wall–soil–indoor air temperatures across greenhouses of varying spans. Over time, all three temperatures exhibited a consistent decreasing trend, with the soil and indoor air temperatures displaying a closely aligned decrease. Beyond 20:30, the temperature hierarchy within greenhouses of different spans was as follows: soil temperature > wall temperature > indoor air temperature.

### 3.6. LSTM Prediction Model

The wall’s heat storage capacity was lower than that of the soil due to lower air temperatures. To precisely control the greenhouse environment and maintain optimal crop growing conditions, this study conducted predictive analyses on air and wall temperatures in both the S1 and S2 greenhouses. Key data points for air and wall temperatures were selected, and a prediction model was developed utilizing the long short-term memory (LSTM) network.

#### 3.6.1. Measurement Point Optimization

**Figure 19:**
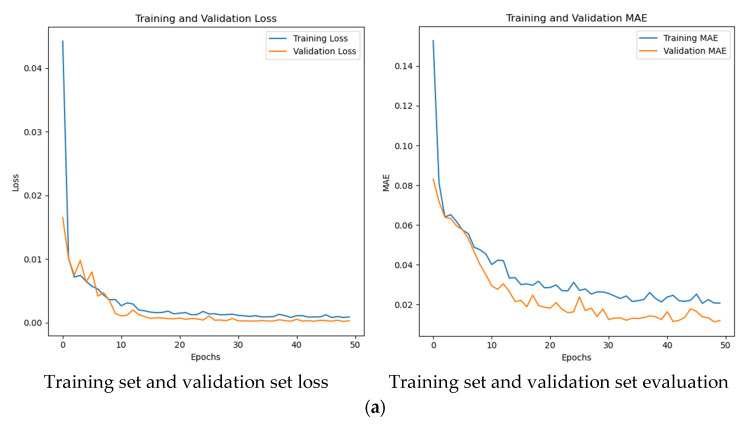

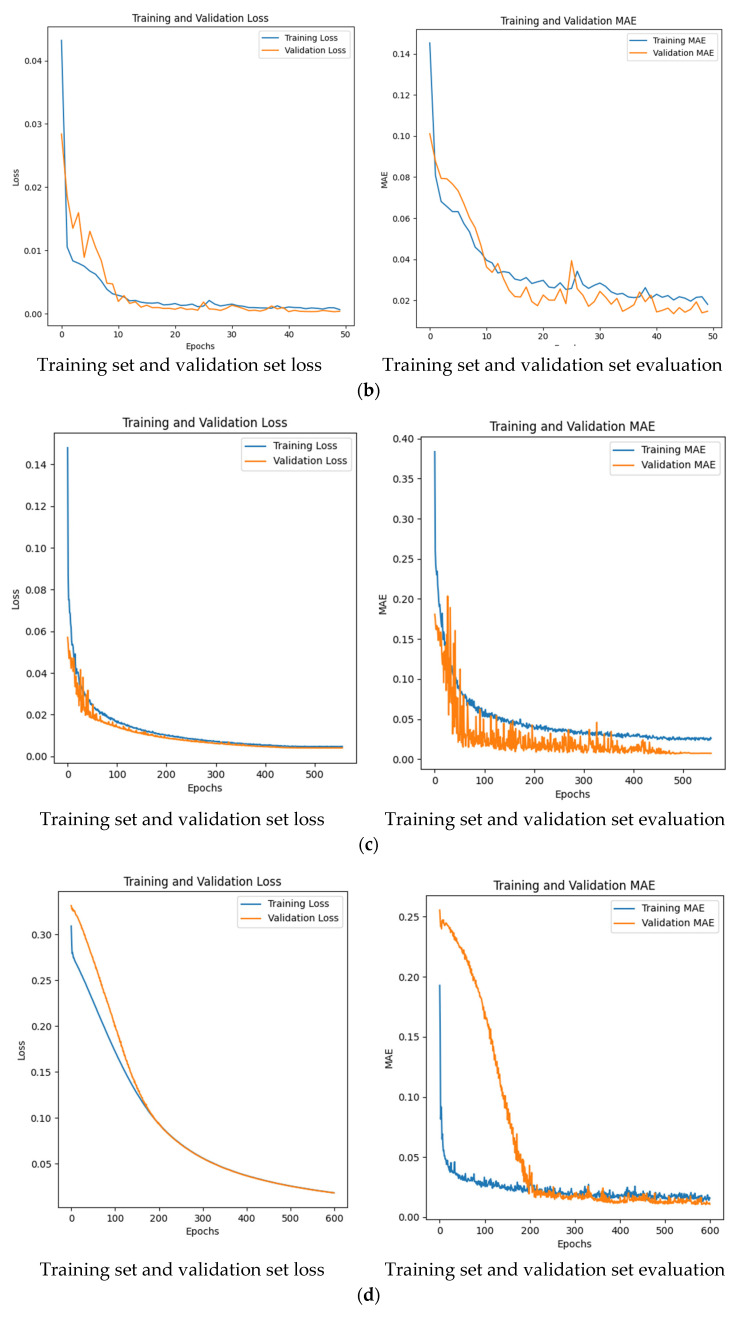
Training set and validation set loss, training set and validation set evaluation. (**a**) The temperature of measurement points W1-5 on the wall of S1 greenhouse; (**b**) the temperature of measurement points W1-5 on the wall of S2 greenhouse; (**c**) the temperature of air measurement points 6-4 in the S1 greenhouse; (**d**) the temperature of air measurement points 6-4 in the S2 greenhouse.

Predictions were conducted for temperatures at critical measurement points (i.e., air and wall temperatures) throughout the overwintering season to establish a scientific foundation for dynamically regulating the greenhouse environment. This aims to maintain stable and optimal environmental conditions conducive to high-quality crop cultivation. The LSTM model was employed to forecast temperatures at the W1-5 and air 6-4 measurement points (X = 7500 mm, Y = 1200 mm) of the W1 section of the indoor wall in the S1 and S2 greenhouses from 28 January 2025 to 3 February 2025. The dataset was divided into a training set (comprising data from 28 January to 2 February 2025) and a test set (incorporating data from 3 February 2025) at a ratio of 7:3. Subsequently, the selected temperature datasets were visualized and analyzed, considering training set loss, validation set loss, training set evaluation, and validation set evaluation within the model. The outcomes are depicted in Figure 19.

The model evaluation indicators, including mean absolute error (MAE), mean square error (MSE), and coefficient of determination (R^2^), were computed for the S1 greenhouse model. The MAEs for wall points W1-5 and air points 6-4 were 0.16 °C and 0.29 °C, respectively, with corresponding MSEs of 0.05 °C and 0.29 °C. Both sets of measurements yielded R^2^ values of 0.99. In the S2 greenhouse model, the MAEs for wall points W1-5 and air points 6-4 were 0.2 °C and 0.3 °C, with MSEs of 0.08 °C and 0.23 °C, respectively, and R^2^ values of 0.99. These errors were all below 0.3 °C, indicating a strong fit of the model. The high R^2^ values suggest accurate prediction of temperature changes in the greenhouse. Consequently, the LSTM model’s greenhouse temperature predictions were utilized for early warning systems to alert growers of extreme temperatures, enabling timely interventions based on the greenhouse conditions.

#### 3.6.2. Analysis of LSTM Prediction Results

##### Wall Temperature Predictions

**Figure 20:**
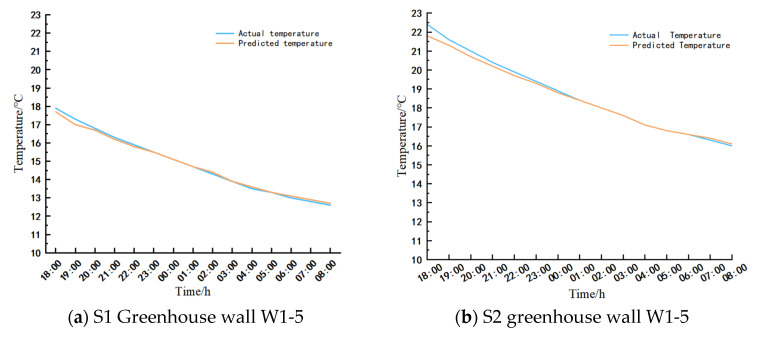
Comparison of wall temperature prediction and test temperature results.

The temperatures predicted for wall measurement points (W1-5) in greenhouses S1 and S2, along with the experimental values recorded between 18:00 on 2 February 2025 and 8:00 on 3 February 2025, were analyzed in comparison to the outputs generated by the LSTM prediction model. The findings are illustrated in Figure 20.

Based on both experimental findings and model projections, a decline in wall temperatures from 18:00 to 8:00 the subsequent day was observed in both greenhouses. The S1 greenhouse exhibited a maximum temperature deviation of 0.4 °C between the observed and predicted temperatures, with a minimal error of 0 °C. Similarly, the S2 greenhouse displayed a maximum temperature deviation of 0.8 °C, with a minimum error of 0 °C, underscoring the model’s precise performance.

##### Greenhouse Air Temperature Prediction

The substantial diurnal temperature fluctuations at the air sampling locations within the solar greenhouse necessitated an extended data collection period to enhance the accuracy of predictive modeling. Therefore, data from 28 January to 8 February 2025 was chosen for analysis.

**Figure 21:**
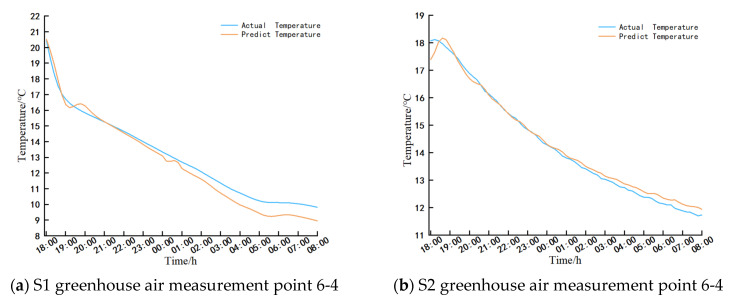
Comparison of air temperature prediction and test temperature results.

Temperature forecasting was conducted for measurement points 6-4 (X = 7500 mm, Y = 1200 mm) in the S1 and S2 greenhouses from 18:00 on 2 February 2025 to 8:00 on 3 February 2025. Experimental data were compared with predictions generated by the LSTM model, and the results are illustrated in Figure 21.

Based on empirical evidence and model predictions, it is evident that the wall temperature of both greenhouses has exhibited a decreasing trend from 18:00 to 8:00 the following day. The S1 greenhouse shows a maximum temperature deviation of 0.7 °C and a minimum error of 0 °C, while the S2 greenhouse displays a maximum temperature deviation of 0.7 °C and a minimum error of 0 °C. These findings underscore the superior performance of the model predictions.

In conclusion, the predictive outcomes for air and wall sensors in various greenhouse structures demonstrate a high level of accuracy. Consequently, the LSTM model proves to be a valuable tool for preemptive monitoring and control of diverse solar greenhouse systems. By forecasting both the timing and magnitude of temperature fluctuations, the LSTM model facilitates precise greenhouse management, thereby optimizing the growing conditions for the crops.

## 4. Conclusions

This study analyzed the synergistic interactions among soil, walls, and indoor air in greenhouses with varying spans (S1 and S2) during the night-time. The analysis was conducted through experimental investigations, K-means classification optimized using the grey wolf algorithm, computational fluid dynamics (CFD) simulations, and long short-term memory (LSTM) predictions.

1. The K-means classification method, optimized using the grey wolf algorithm, was employed to analyze the night-time temperatures of indoor air in greenhouses S1 and S2. The low-temperature boundary point in indoor air was found to be at a height of 1200 mm with a span of 7500 mm in both greenhouses. Notably, the average temperature of the low-temperature boundary point in greenhouse S2 was markedly higher than that in greenhouse S1.
2. The nocturnal soil temperature analysis in greenhouses S1 and S2 employed a cubic fitting function, assuming a consistent temperature at a depth of 300 mm below the soil surface. The soil temperature range in greenhouse S1 was narrower than that in S2. The soil in each greenhouse was segmented vertically into two zones: 0 mm < y < −150 mm and −150 mm < y < −600 mm. Horizontally, the soil was categorized into three zones: low-temperature, constant-temperature, and high-temperature regions.
3. The heat transfer occurred from the outer surface of the solar greenhouse wall at 0 mm to the inner surface at 0 mm and at a horizontal depth of 200 mm. Steady-state wall temperatures were observed for greenhouses S1 and S2 at heights exceeding 400 mm from the inner wall surface. The steady-state boundary for the exterior wall was identified at a horizontal depth beyond 200 mm. The placement of the quilt notably influenced the vertical temperature distribution within the greenhouse wall.
4. At the low temperature of the S1 greenhouse, an investigation was carried out on the combined temperature variations of soil, walls, and indoor air. This analysis revealed four distinct categories through K-means classification optimized by the grey wolf algorithm: high-temperature region, medium-high temperature region, medium-low temperature region, and low-temperature region. The soil and walls were categorized under the high-temperature region. The medium-high temperature region spanned X = (3000, 9000) mm in width and Y = (0, 1200) mm in height. The medium-low temperature region spanned X = (6000, 9000) mm in width and Y = −(1200, 4000) mm in height. Lastly, the low-temperature region spanned X = −(800, 3000) mm in width and Y = −(150, 1200) mm in height.
5. The computational fluid dynamics (CFD) simulations effectively depicted the synergistic variation in cloud cover patterns within the S1 and S2 solar greenhouses operating under identical conditions. Utilizing an LSTM model, accurate predictions were made for the W1-5 and air 6-4 measurement points at coordinates X = 7500 mm and Y = 1200 mm within the W1 section of the greenhouse walls in both S1 and S2.

## 5. Discussion

This study extends the prior research by integrating experimental studies, GWO-K-means classification, CFD simulation, and LSTM prediction to investigate the thermal conditions within solar greenhouses. It elucidates the boundary characteristics of the low-temperature zone (height: 1200 mm, span: 7500 mm) and the steady-state layer of the wall (indoor > 400 mm, outdoor > 200 mm). This study introduces a zoning control strategy, dividing the greenhouse into four temperature zones, offering a theoretical framework for greenhouse design and intelligent control. Nonetheless, limitations arise from the constraints in experimental resources and minor variations in quilt positioning and ventilation systems between the two greenhouses. Subsequent research will incorporate biological indicators such as chlorophyll fluorescence and root water uptake rate to explore the correlation between temperature zoning and crop stress, with the goal of optimizing greenhouse design and management to improve crop yield and quality.

While the experimental data and model analysis demonstrate temperature change patterns among soil, walls, and indoor air, these patterns do not inherently imply a definitive “synergistic mechanism”. The observed changes likely result from intricate heat and mass exchange interactions, necessitating additional quantitative scrutiny for confirmation. Subsequent investigations will prioritize quantifying the heat and mass flow between these components to better elucidate their interplays. This will be pursued through enhanced experimental methodologies and sophisticated numerical simulations.

## Figures and Tables

**Table 1:** Arrangement of greenhouse air measurement points.

			Measurement Line Number					
		1	2	3	4	5	6	7
	X/m	800	1500	3000	4500	6000	7500	9000
Measurement point number	1	0/0	−/0	−/0	−/0	−/0	−/0	−/0
	2	0.3/0.3	0.3/0.3	0.3/0.3	0.3/0.3	0.3/0.3	0.3/0.3	0.3/0.3
	3	0.6/0.6	0.6/0.6	0.6/0.6	0.6/0.6	0.6/0.6	−/0.6	0.6/0.6
	4		1.2/1.2	1.2/1.2	1.2/1.2	1.2/1.2	1.2/1.2	1.2/1.2
	5			1.8/1.8	1.8/1.8	1.8/1.8	1.8/1.8	1.8/1.8
	6			2.4/−	2.4/2.4	2.4/2.4	2.4/2.4	2.4/2.4
	7				3.0/−	3.0/3.0	3.0/3.0	3.0/3.0
	8					3.6/−	3.6/3.6	3.6/3.6
	9							4.0/4.0

**Table 2:** Arrangement of soil temperature measurement points in daylight greenhouse (unit: mm).

Coordinates	Measurement Point Number											
	S1	S 2	S 3	S 4	S 5	S 7	S 8	S9	S 10	S 11	S 12	S 13
X	400	800	1200	1600	2000	3000	4500	7500	7800	8200	8600	9000
Y	−150	−150	−150	−150	−150	−150	−150	−150	−150	−150	−150	−150

**Table 3:** Arrangement of greenhouse wall temperature measurement points (unit: mm).

Cross-Section	Wall Cross-Section Height	Measurement Point Number				
		1	2	3	4	5
W1	3200/3600	0	200	400	600	800
W2	2400/2700	-	200	-	-	-
W3	1600/1800	0	200	400	600	800
W4	800/900	-	200	-	-	-
W5	0/0	0	200	400	600	800

**Table 4:** Analysis of variance (ANOVA).

		Sum of Squares	Degrees of Freedom	Mean Square	F Statistic	*p*-Value
22:00	Between Groups	3.93	2	1.97	8.57	0.003
	Within Groups	3.19	14	0.23		
	Total	7.12	16			
02:00	Between Groups	3.05	2	1.53	5.28	0.020
	Within Groups	4.11	14	0.29		
	Total	7.16	16			
06:00	Between Groups	2.33	2	1.17	4.03	0.041
	Within Groups	4.04	14	0.29		
	Total	6.37	16			

**Table 5:** Initial boundary temperature values.

					(**a**)						
**Location**	**A1**	**A2**	**A3**	**A4**	**A5**	**S1**	**S2**	**S3**	**S4**	**S5**	**S6**
**S1 Greenhouse Initial Temperature (°C)**	16.0	16.1	17.1	17.7	16.7	17.6	19.5	20.2	16.7	18.6	19.3
					(**b**)						
**Location**	**W1**	**W2**	**W3**	**W4**	**W5**	**W6**	**WO-1**	**WO-2**	**North Wall Thermal Zone**		
**S1 Greenhouse Initial Temperature (°C)**	16.5	10.7	18.0	12.1	24.9	18.4	−4.3	−1.7	13.4		

**Table 6:** Thermal physical properties of materials.

Parameters	Density kg/m^3^	Specific Heat Capacity J/(kg·K)	Thermal Conductivity W/(m·K)	Absorption Coefficient	Scattering Coefficient	Refractive Index
PE film	950	1600	0.34	0.15	0	1.72
Soil wall	2000	1050	0.8	0.88	0.12	1.92
Soil	1600	1050	0.75	0.88	0.12	1.92
Back slope	600	2500	0.88	0.7	0	1.72
Heat Preservation Quilt	70	1880	0.12	0.1	0	1.72
Crop	560	2100	0.19	0.35	0.1	2.77

**Table 7:** Categorization of height hypothermia for different spans.

Span of a Measurement Line x/mm	800	1500	3000	4500	6000	7500	9000
S1 Cryogenic Boundary Point Height y/mm	600	1200	1200	1200	1200	1200	2400
S2 Cryogenic Boundary Point Height y/mm	300	600	1200	1200	1200	1800	1200
S1 Low-Temperature Boundary Point Mean Temperature/°C	8.2	8.4	8.7	8.8	9	9	9.8
S2 Low-Temperature Boundary Point Mean Temperature/°C	10.1	11.9	11.9	12.1	12.3	12.6	12.9
S1 Low-Temperature Boundary Point Minimum Temperature/°C	6.7	6.8	7.1	7.3	7.4	7.4	8.1
S2 Low-Temperature Boundary Point Minimum Temperature/°C	8.6	10.7	10.6	10.7	11.0	11.3	11.6

**Table 8:** Categorization of spanning cryogenics at different heights.

High Degree y/mm	300	600	1200	1800	2400	3000	3600
S1 Cryogenic boundary point span x/mm	3000	4500	7500	7500	6000	7500	7500
S2 Cryogenic boundary point span x/mm	800	800	6000	7500	7500	7500	7500
S1 Low-Temperature Boundary Point Mean Temperature/°C	9.1	8.8	9.0	9.3	9.5	10.0	9.4
S2 Low-Temperature Boundary Point Mean Temperature/°C	10.1	10.7	12.3	12.6	12.6	12.4	12.6
S1 Low-Temperature Boundary Point Minimum Temperature/°C	7.5	7.2	7.4	7.5	7.8	8.4	7.8
S2 Low-Temperature Boundary Point Minimum Temperature/°C	8.6	9.3	11	11.3	11.3	11.1	11.2

**Table 9:** Steady-state boundary points for indoor wall temperatures.

Depth of the Temperature Steady State Boundary Point of the Solar Greenhouse Wall from the Surface Level of the Wall. Unit: mm				
	cross-section	W1	W3	W5
solar greenhouse				
sunken solar greenhouse (S1)		200	400	400
conventional solar greenhouse (S2)		400	200	400

## Data Availability

Data are contained within the article.
